# Predictability of a Mandibular Corpus Length Multivariate Model Integrating Björk-Jarabak Measurements and the Cephalometric Norm

**DOI:** 10.7759/cureus.87864

**Published:** 2025-07-13

**Authors:** María Eugenia Balderas-González, Luis Pablo Cruz-Hervert, Silvia Paulina Martínez-Contreras, Valentina García-Lee, José David Ortiz-Sánchez, Jacqueline Adelina Rodríguez-Chávez, María Eugenia Jiménez-Corona, Gisel García-García, Jeta Kiseri-Kubati, Sergio Sánchez-García

**Affiliations:** 1 Orthodontics, Universidad Cuauhtémoc, San Luis Potosí, MEX; 2 Division of Graduate Studies and Research, Faculty of Dentistry, Universidad Nacional Autónoma de México, Mexico City, MEX; 3 Department of Epidemiology, Instituto Nacional de Cardiología Ignacio Chávez, Mexico City, MEX; 4 Orthodontics, Escuela Nacional de Estudios Superiores Unidad León, León, MEX; 5 Department of Integral Dental Clinics, University Center for Health Sciences, Universidad de Guadalajara, Guadalajara, MEX; 6 Epidemiological Research and Health Services Unit, Aging Area, Instituto Mexicano del Seguro Social, Mexico City, MEX; 7 Orthodontics, University for Business and Technology, Pristina, ALB

**Keywords:** anterior cranial base, björk polygon, cbct, cephalometric tracing, mandibular corpus length, mexican population, non-caucasic population, posterior cranial base, ramus height

## Abstract

Introduction

The cephalometric norm of the mandibular corpus length (MCL) or the one-to-one ratio of the MCL to the anterior cranial base length (ACBL) are cephalometric indicators with unknown predictive capacity and clinical utility. Multivariate regression models enable the use of two or more variables to estimate an expected value, in this case, for MCL. This study compares three approaches to predicting MCL in adults by applying Björk-Jarabak measurements: (i) conventional angular norms, which have limited standalone value; (ii) simple linear‐proportion indices of craniofacial structures; and (iii) a multivariate model that integrates both linear and angular measurements.

Methods

A cross-sectional study was conducted using 100 adult cone beam computed tomography (CBCT) scans (63% female, mean age 29.5 ± 8.4 years) who met strict inclusion criteria. Seven simple linear regression models were analyzed for individual cephalometric variables: ACBL, posterior cranial base length (PCBL), ramus height (RH), saddle angle (SA), articular angle (AA) and gonial angle (Gon), and cephalometric norm adjusted for sex and age. Subsequently, a comprehensive multivariate model was developed. Regression coefficients (β), 95% confidence intervals (95% CI), and determination coefficients (R²) were reported for each model.

Results

Linear measurements revealed a statistically significant association with MCL: anterior cranial length (β = 0.86; 95% CI: 0.72-0.99; R² = 0.6462), posterior cranial length (β = 1.16; 95% CI: 0.92-1.40; R² = 0.5331), and RH (β = 0.84; 95% CI: 0.68-0.99; R² = 0.5757). In contrast, the cephalometric norm had low explanatory power (β = 4.72; 95% CI: -0.47--9.93; R² = 0.1091), and the angular measurements were not significant. The final multivariate model, including the three linear variables, showed a superior predictive capacity (R² = 0.8689), with the following coefficients: ACBL (β = 0.41), PCBL (β = 0.49), RH (β = 0.20), and Gon (β = -0.18; all p <0.01).

Conclusion

These findings suggest that linear craniofacial measurements have greater predictive capacity for the MCL than do norms or angles. The multivariate model increased the explanatory capacity by 22.27% relative to the best individual model. The integration of these variables allows more precise and personalized estimates in adults. The use of multivariate models in MCL clinical practice and their validation in other populations is recommended.

## Introduction

A fundamental goal in orthodontic and maxillofacial diagnosis is to obtain an accurate estimate of the length of the mandibular body [1,2]. This measurement allows us to identify whether a certain skeletal classification is associated with mandibular size alterations, either due to excessive or deficient growth or to deviations in its developmental direction or spatial relationship to other cranial structures. In this context, the availability of tools that allow a reliable and objective estimation of said length is crucial for correct clinical decisions [1-4].

Over the years, various methods have been proposed attempting to provide reference parameters to estimate mandibular length. Among the most widely used methods are cephalometric norms, the proportional relationship between the mandibular body and the anterior cranial base, and statistical models based on multiple linear regression [2,4-8]. While these approaches have been widely applied, each has specific limitations regarding its accuracy and clinical applicability, which highlights the need for more rigorous comparative evaluations.

The most widespread method in clinical practice has been the use of cephalometric norms, which are based on average values and standard deviations, establishing a range considered "normal" [1,9]. Although this approach may be useful as an initial point of reference, its main disadvantage lies in its poor adaptability to populations different from those where the normative data originated [3,10]. This raises important questions about its validity as a universal predictive tool [11].

Within this same traditional approach, the Björk-Jarabak analysis suggests an approximately one-to-one proportional relationship between the length of the mandibular body and the anterior cranial base [1,12,13]. Its operational simplicity and reliance on a relatively stable anatomical structure, such as the anterior cranial base, offer a certain advantage over other methods. However, its practical application yields inconsistent results [10,11], and its utility as a predictive estimator has not yet been formally validated. Moreover, it remains unclear whether this parameter outperforms other craniofacial structures, such as the posterior cranial base or the mandibular ramus height, in the precise estimation of the mandibular corpus length (MCL) [12,13].

Therefore, it is necessary to establish methods to compare measurements between reference landmarks that are often considered clinically reliable, despite limited empirical validation supporting their accuracy. Discrepancies observed when certain indicators are applied may be due to insufficient analysis of which variables truly contribute to the estimation model. Thus, it is important to evaluate not only the proportionality between structures but also their ability to accurately predict the ideal mandibular length [14].

In recent years, proposals have emerged that incorporate more complex statistical tools, such as multivariate linear or multilevel regression models [15-18]. These methods allow simultaneous evaluation of the impact of multiple cephalometric variables, providing more precise estimates of the expected value. One of its main strengths is their ability to identify which structures have a statistically significant contribution and which, despite their theoretical relevance, do not contribute to the predictive model [2,6,19,20]. However, because of its technical complexity, their application in clinical practice remains limited [2,4,5,21]. Even so, they represent a promising alternative to establish personalized norms based on combinations of traditionally recognized linear and angular cephalometric variables, such as those that found the Björk-Jarabak analysis [22,23].

In this scenario, it is essential to determine the agreement and explanatory power among different estimation methods to identify which yields greater precision for mandibular length estimation, particularly in specific populations such as young adults. Furthermore, it has not yet been determined whether structures such as the posterior cranial base, the ramus height (RH) or certain cephalometric angles, such as the saddle (SA), articular angle (AA) or gonial angle (Gon), can offer a better estimate than the traditional anterior cranial base [12]. Likewise, it is unknown whether the articular analysis of these variables can optimize existing models, reduce overestimation or underestimation, and thus improve the clinical utility of cephalometric diagnosis.

This study compares three strategies for predicting MCL: (i) traditional angular norms, whose standalone value is limited; (ii) simple linear-proportion indices of craniofacial structures; and (iii) a multivariate model that integrates both linear and angular measurements. We hypothesize that the multivariate approach, by capturing several complementary dimensions of craniofacial morphology, will yield greater diagnostic accuracy and clinical usefulness than either angular norms or linear proportions alone.

## Materials and methods

A cross-sectional study was conducted using the tomographic records of adult patients attending the orthodontic clinic of Cuauhtémoc University at San Luis Potosí Campus in Mexico. Sample selection was performed via simple randomization, considering a total population of 422 cone beam tomographies (CBCTs) from the records spanning the period of 2021-2023, which was carried out via Epidat program version 4.2. Ethical protocols were followed during data collection. AII informed consent forms were previously signed by patients and were integrated into patients' files, granting authorization for the use of their records for academic and research purposes. The protocol was approved by the ethics and research committee of Cuauhtémoc University, San Luis Potosí, Mexico (No. CEI-UCSLP-24-004).

The inclusion criteria were as follows: 1) adults between 18 and 50 years of age; 2) absence of a history of previous orthodontic treatment; and 3) not having any type of syndrome, malformation or disease potentially affecting jaw development. The exclusion criteria were as follows: 1) surgical patient records; 2) dental agenesis, including teeth, supernumerary or with micro- or macrodontia; and 3) tomographic findings of any alterations that prevent adequate evaluation.

Sample size

To reduce selection bias and improve statistical efficiency, the required number of CBCTs was first estimated with a formal sample-size calculation, thereby minimizing the risk of type I and type II errors. The minimum sample size was estimated to be 97 CT scans. The above value was calculated via an online sample size calculator via the A-priori Sample Size Calculator for Multiple Regression module [24], which considers an anticipated effect of 0.15, a power of 0.80, an alpha of 0.050, and six predictor variables. From a total of 422 CBCTs, 322 were initially drawn at random, and from these, we randomly included 100 according to the initial sample calculation for analysis.

Variables

The cephalometric variables are classified as linear measurements (mm) and angular measurements (°): linear measurements comprise the MCL, the anterior and posterior cranial base lengths (ACBL and PCBL), and the RH; angular measurements comprise the AA, the SA, and the Gon. Sociodemographic covariables of age and sex were obtained from medical records. The variables are described in Table 1.

**Table 1:** Variable Description for Cephalometric Norms of Björk-Jarabak Analysis ACBL: anterior cranial base length, PBCL: posterior cranial base length, RH: ramus height, MCL: mandibular corpus length, AA: articular angle, SA: saddle angle, Gon: gonial angle

Linear measurements		Definition
ACBL	Anterior cranial base length	Distance between landmark Nasion and landmark Sella (S-N)
PCBL	Posterior cranial base length	Distance between landmark Sella and landmark Articulare (S-Ar)
RH	Ramus height	Distance between landmark Articulare and landmark Gonion (Ar-Go)
MCL	Mandibular corpus length	Distance between landmark Gonion and landmark Gnathion (Go-Gn)
	Angular measurements	
AA	Articular angle	Angle formed between Sella-Articulare-Gonion (S-Ar-Go)
SA	Saddle angle	Angle formed between Nasion-Sella-Articulare (N-S-Ar)
Gon	Gonial angle	Angle formed between Articulare-Gonion-Gnathion (Ar-Go-Gn)

Predictability was measured as follows: 1) The coefficient of determination of the regression models (R2), which represents the percentage of explanation of each model for the estimation of MCL. The values of R2 range from 0 to 1.0, where 1 represents 100% of the explanatory capacity of the phenomenon. 2) The difference between the actual and estimated values of MCL.

Data collection

**Figure 1:**
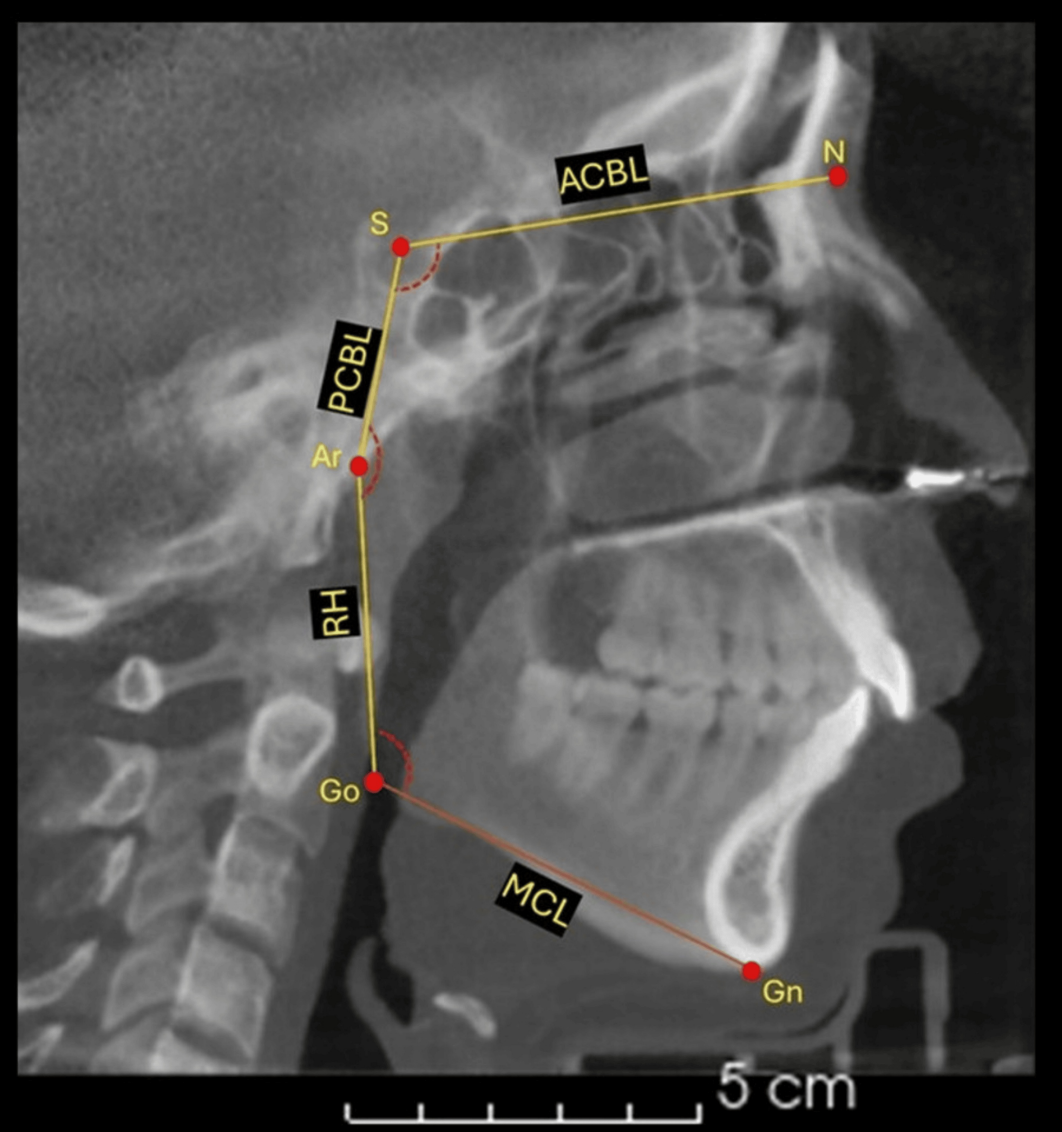
Identification of the cephalometric variables in the lateral radiograph. The image of the lateral radiograph obtained from cone beam tomography in orthogonal projection is shown, that is, without distortion of the structures. The image illustrates the cephalometric tracings made via artificial intelligence via the WebCeph online program. ACBL: anterior cranial base length, PBCL: posterior cranial base length, RH: ramus height, MCL: mandibular corpus length, N: nasion, S: sella, Ar: articulare, Go: gonion, Gn: gnathion

All CBCTs were performed via the NEWTOM® VGi tomograph (NewTom, Imola, Italy) with the following parameters: 110 kV, 1-20 mA pulsed mode, 18-26 seconds of scanning time, and a large field of view (FOV) of 15x15 cm. The CBCT examination was performed with the patient in a standing position. After the X-ray scan, the DICOM image files were processed with NNT software. 3D Slicer Software version 5.2.2 (www.slicer.org) was used to obtain the projection of the lateral skull radiograph in orthogonal projection to guarantee the 1-1 evaluation of the structures and with a calibration rule. The JPG images were analyzed with WebCeph® (v 1.0, Asma Software, Seoul, Korea) AI software, as illustrated in Figure 1. After each scan was uploaded to the platform, it was manually calibrated with the on-screen ruler. The software then used its AI engine to identify all radiographic landmarks and perform the Jarabak cephalometric analysis automatically, without further operator editing. Finally, WebCeph exported the resulting linear and angular measurements to Excel (Microsoft, Redmond, WA, USA), from which the study database was constructed.

Data quality

WebCeph has consistently shown good-to-high reliability, as reported by other studies [25-27]. Nevertheless, to assess measurement consistency, we conducted a pilot study on 10 CBCT scans outside the main sample, tracing each scan twice in WebCeph as independent observations. Intraclass correlation coefficients [28] (ICC; two-way random-effects, consistency) confirmed high landmark reproducibility overall: RH and the Gon showed good reliability (ICC ~0.80); the ACBL, PCBL, MCL, and AA achieved excellent reliability (ICC > 0.90); whereas the SA displayed only moderate agreement (ICC = 0.42).

Statistical analysis

A descriptive analysis of all variables was performed, reporting the mean, standard deviation (SD), minimum and maximum. To estimate the length of the mandibular body, seven statistical models were performed: six univariate linear regression models were first fitted-one for each cephalometric variable-followed by a multivariate model that initially included all variables; using a stepwise approach, only variables with a Wald-test p-value < 0.200 were retained, resulting in a reduced model selected based on plausibility and model-validation criteria. To assess each model, we estimated the regression coefficients (Coef.) and their 95% confidence intervals (95% CI); we then calculated the differences between the observed and predicted mandibular body measurements through residual analysis and plotted them in a dot-plot graph, indicating the 25th (Q1), 50th (median), and 75th (Q3) percentile, along with any outlier values, for each method. The nonparametric Friedman test was selected to evaluate the differences in medians between the residuals between all the models since the measurements correspond to repeated data per individual and compliance with the normality and homoscedasticity assumptions required by traditional ANOVA is not guaranteed.

## Results

A total of 100 lateral skull radiographs were analyzed. Sixty-three percent of the images corresponded to women, and 37% corresponded to men. The average age of the participants was 29.5 years, with a standard deviation (SD) of 8.4 years.

Regarding the cephalometric measurements, the general mean MCL was 70.3 mm (SD 10.5), with a range between 57.3 mm and 121 mm. When the data were disaggregated by sex, the average length was 67.7 mm (SD 6.9) in women and 74.8 mm (SD 13.7) in men. Table 2 presents the descriptive statistical values for the cephalometric variables evaluated.

**Table 2:** Results of the descriptive analysis of the Björk-Jarabak cephalometric variables.

Variable	n	Mean	SD	Min	Max
Ratio Mandibular corpus length to anterior cranial base length	100	1.1	0.1	0.9	1.6
Anterior cranial base length (mm)	100	65.8	9.6	44.3	112.2
Posterior cranial base length (mm)	100	33.6	6.2	24.2	64.1
Ramus height (mm)	100	48.9	9.2	34.2	93.4
Mandibular corpus length (mm)	100	70.3	10.5	57.3	121
Articular angle (Degrees)	100	146.5	8.6	124.3	172.2
Saddle angle (Degrees)	100	126.5	7.4	108.3	141.1
Gonial angle (Degrees)	100	123.9	6.6	109.5	143.2

Multivariate model of multivariate regression by cephalometric characteristics for the prediction of MCL

Seven simple linear regression models were constructed, each corresponding to a cephalometric variable: the cephalometric norm, ACBL, PCBL, RH, SA, AA and Gon angles. All models were initially adjusted for age and sex.

The results revealed that three linear measurements presented a statistically significant association with the length of the mandibular body: the anterior cranial length (Coef. = 0.86; 95% CI: 0.72-0.99; p <0.001), the posterior cranial base (Coef. = 1.16); 95% CI: 0.92-1.40; p <0.010) and the RH (Coef. = 0.84; 95% CI: 0.68-0.99; p <0.001). In contrast, the models that included the cephalometric norm and the angular measurements did not reach statistical significance. Notably, in the latter, sex was significantly associated, whereas age was not significantly associated in any model (Table 3).

**Table 3:** Results of the multivariate linear regression models. Coefficient = linear regression coefficient; 95%CI = 95% confidence interval; *=p<0.050; **=p<0.010; ***=p<0.001

Variable	Model 1	Model 2	Model 3	Model 4	Model 5	Model 6	Model 7	Model 8
	Cephalometric Norm	Anterior Cranial Base	Posterior Cranial Base	Ramus Height	Saddle angle	Articular Angle	Gonial Angle	Integral model
	Coefficient	Coefficient	Coefficient	Coefficient	Coefficient	Coefficient	Coefficient	Coefficient
	(95%CI)	(95%CI)	(95%CI)	(95%CI)	(95%CI)	(95%CI)	(95%CI)	(95%CI)
Cephalometric Norm	4.72	----------	----------	----------	----------	----------	----------	----------
	(-0.47 to 9.93)							
Anterior Cranial Base	----------	0.86 ***	----------	----------	----------	----------	----------	0.41 ***
		(0.72 to 0.99)						(0.30 to 0.51)
Posterior Cranial Base	----------	----------	1.16 ***	----------	----------	----------	----------	0.49 ***
			(0.92 to 1.40)					(0.49 to 0.81)
Ramus Height	----------	----------	----------	0.84 ***	----------	----------	----------	0.20 **
				(0.68 to 0.99)				(0.08 to 0.32)
Saddle angle	----------	----------	----------	----------	-0.07	----------	----------	----------
					(-0.34 to 0.19)			
Articular Angle	----------	----------	----------	----------	----------	-0.11	----------	----------
						(0.35 to 0.11)		
Gonial Angle	----------	----------	----------	----------	----------	----------	-0.25	-0.18 ***
							(-0.56 to 0.04)	(-0.33 to -0.02)
Sex	-6.08 **	-1.07	-3.28	-1.9	-7.01 **	-6.99 **	-6.84 **	----------
	(-10.30 to -1.85)	(-0.16 to 0.13)	(-6.34 to -0.22)	(-4.88 to 1.07)	(-11.16 to -2.86)	(-11.12 to -2.86)	(-10.94 to -2.74)	
Age	0.01	0.01	-0.02	0.042	-0.001	0.001	0.01	----------
	(0.72 to 0.99)	(0.72 to 0.99)	(-0.19 to 0.14)	(-0.12 to 0.20)	(-0.24 to 0.23)	(-0.23 to 0.23)	(-0.22 to 0.24)	
Constant	78.83 ***	15.95***	37.13***	31.00***	91.64***	99.03***	113.16***	47.53 ***
	(68.83 to 88.83)	(3.85 to 28.06)	(25.69 to 48.64)	(19.47 to 42.54)	(56.35 to 126.94)	(63.73 to 134.32)	(75.36 to 150.96)	(30.07 to 65.00)

Comprehensive multivariate model for the prediction of MCL

Initially, a saturated multivariate model was formulated to include all linear and angular measurements, excluding the cephalometric norm. Using the stepwise method, a reduced model was subsequently constructed with statistically significant variables.

The final model included the length of the ACBL (Coeff. = 0.53; 95% CI: 0.38-0.68; p <0.001), PCBL (Coeff. = 0.44; 95% CI: 0.24-0.65; p <0.001), and the RH (Coeff. = 0.28; 95% CI: 0.12-0.43; p <0.010). Age and sex were evaluated in the full model but were excluded from the final model because they showed no statistically significant association with mandibular corpus length and did not act as effect modifiers or confounders. Neither age nor sex were included in the final model, since they did not show statistical significance. This model aims to optimize the prediction of MBL, improving upon previous understanding through identification of independently associated factors (Table 3).

Application of MCL estimation - integral model (Model 8)

Below, we provide an example demonstrating the application of the multivariate linear regression equation from Model 8 to estimate a value. For this illustration, we use the coefficients reported in Model 8 for each variable, along with hypothetical values assigned to the independent variables (x). This stepwise substitution clearly illustrates how each measurement, multiplied by its respective coefficient, and the constant term combine to yield the final predicted mandibular corpus length:

MCL = β₀ + βACBL × ACBL + βPCBL × PCBL + βRH × RH + βGon × Gon

Using the equation using the following terms and coefficients: 1) β₀ (Constant) = 47.53; 2 βACBL = 0.41; βPCBL = 0.49, βRH = 0.20, and βGon = -0.18

If we consider, for example, a hypothetical case, the substitution with provided values is stated as follows:

RH = 55 mm, will be multiplied by βRH = 0.20;

ACBL = 70 mm, will be multiplied by βACBL = 0.41;

PCBL = 36 mm, will be multiplied by βPCBL = 0.49;

Gon = 30°, will be multiplied βGon = -0.18;

Step 1: MCL = 47.53 + 0.41 × 70 + 0.49 × 36 + 0.20 × 55-0.18 × 30

Step 2: = 47.53 + 28.70 + 17.64 + 11.00-5.40

Step 3: = 99.47 mm

Result = MCL: ~ 99.5 mm.

Explanatory capacity of multivariate models for estimating MCL

The predictive capacity of each model was evaluated via the coefficient of determination (R²). The model based on the cephalometric norm had the lowest explanatory value (R² = 0.1091), which represents only 10.9% of the variance in MCL. In contrast, the models based on linear variables presented higher explanatory values: 64.62% for the ACBL (R² = 0.6462), 53.31% for the PCBL (R² = 0.5331) and 57.57% for the RH (R² = 0.5757).

**Figure 2:**
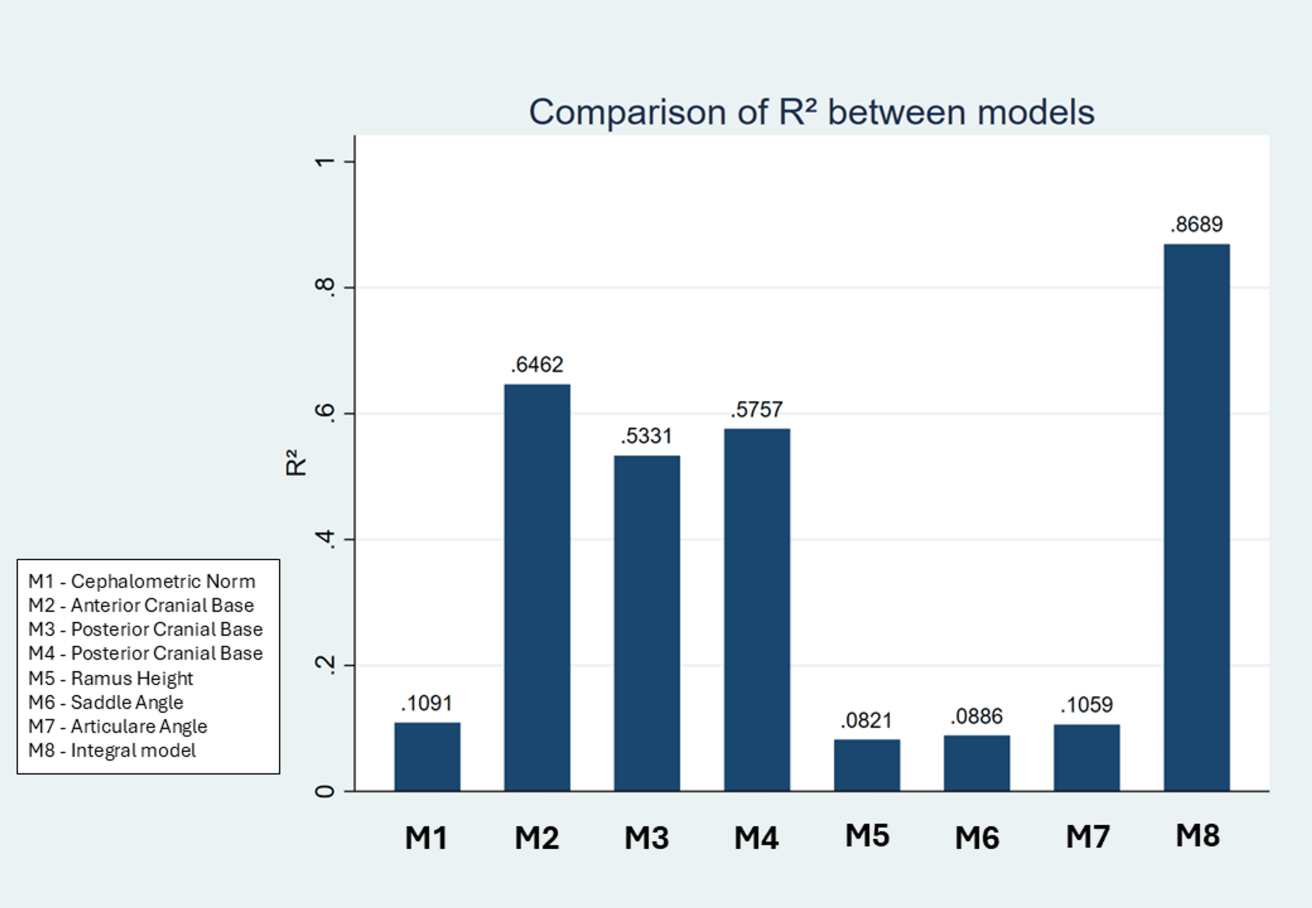
Comparison of the R2 values of all multivariate models. R² = coefficient of determination; higher values indicate greater explanatory power of the model. The models labeled in the figure include combinations of ACBL, PCBL, RH, and Gon. * p <0.05 for all coefficients of the model; n = 100 CBCTs of adults (63% women, mean age = 29.5 ± 8.4 years). ACBL: anterior cranial base length, PBCL: posterior cranial base length, RH: ramus height, Gon: gonial angle, CBCT: cone beam computed tomography

By integrating these three linear variables, the multivariate model reached an explanatory capacity of 86.89% (R² = 0.8689), which represents an increase of 22.27% with respect to the univariate model with greater predictive capacity. This improvement suggests that the combination of multiple linear measurements is more effective than their isolated analysis (Figure 2).

Evaluation of residuals between multivariate models

**Figure 3:**
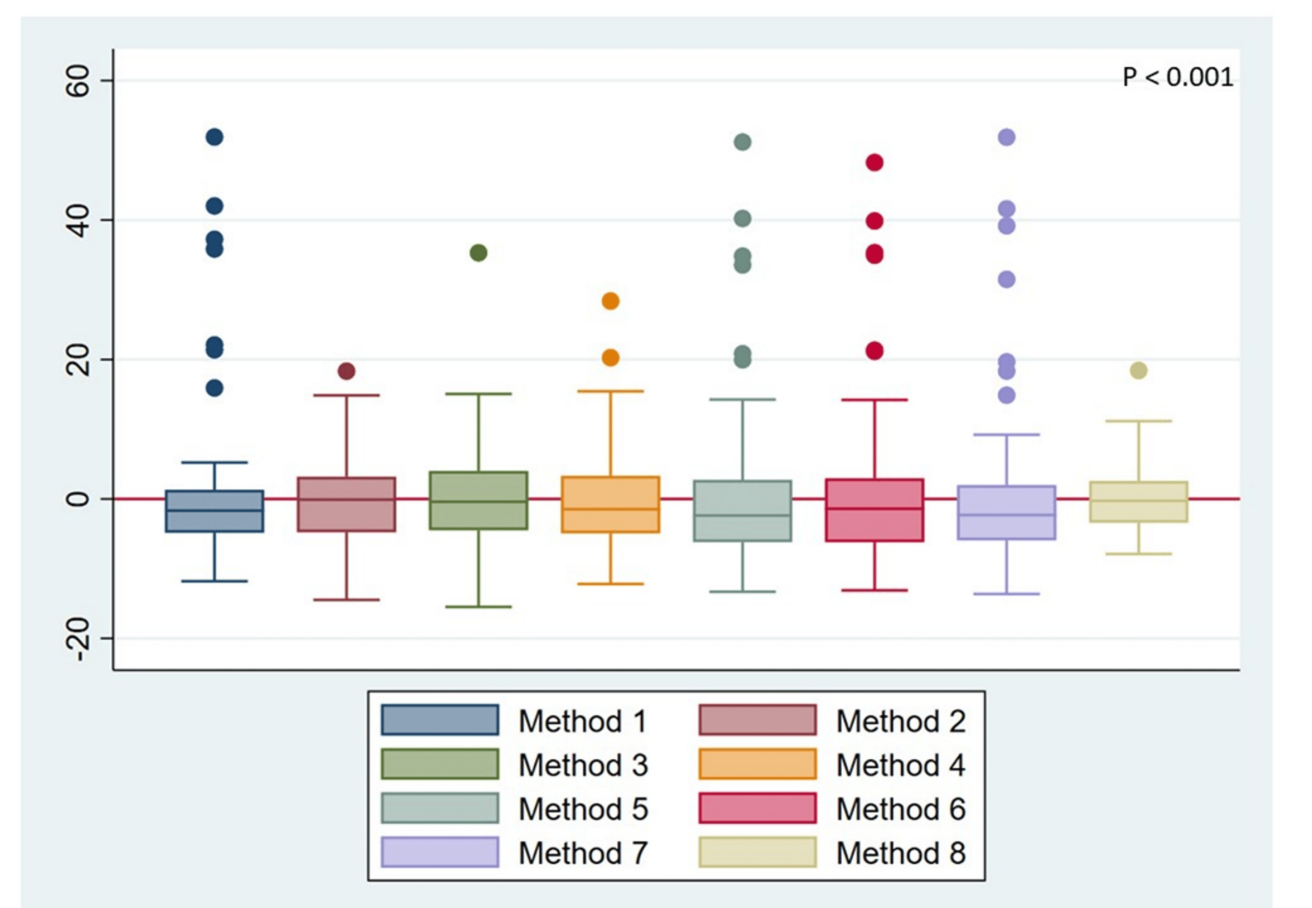
Box and whisker plots to observe the behavior of the residuals of each model. Each box shows the interquartile range (IQR) of the residuals; the center line marks the median. Whiskers extend to 1.5 × IQR or the nearest extreme value, and points outside this range are considered outliers. A narrower IQR and a median close to zero indicate greater precision and lower model bias.

The difference between the real values and the estimated values of the MCL was evaluated using analysis of residues and box plot graphical representation (Figure 3). In this graph, the red horizontal line represents the optimal point (residual = 0), indicating no difference between the actual and estimated measurements.

Notable differences were observed between the methods. A positive residual indicated an underestimation, whereas a negative value suggested an overestimation of mandibular length. The boxes that showed less dispersion-and thus greater precision-corresponded to the multivariate model and the cephalometric norm. However, despite its apparent precision, the standard presented a low R² value, limiting its predictive utility (Figure 3).

The multivariate model stood out as the most reliable, revealing less variability and fewer outliers, and its median coincided with the optimal value. These findings reinforce its diagnostic utility to estimate the MCL. Consequently, the integration of multiple cephalometric measurements in multivariate models can optimize the estimation of MCL in adult populations.

## Discussion

The findings of this study provide insight to the clinical predictability of MCL via different estimation methods, particularly the traditional measurements of the Björk-Jarabak analysis and the cephalometric norm, in comparison to multivariate statistical models. Although it has been assumed that the one-to-one ratio between the MCL and the ACBL represents an ideal morphological pattern, our results show that this criterion, although useful as a general point of reference, lacks sufficient predictive capacity when evaluated in isolation. This behavior is consistent with evidence that the morphology of the cranial base, especially the anterior length (sella ‑ nasion), the posterior length (sella ‑ articulare) and the basal flexion, influences anteroposterior mandibular development; shorter and less flexed cranial bases are characteristic of subjects with retruded mandible, whereas greater flexion is associated with longer and prognathic mandible [12].

The analysis of the simple linear regression models revealed that the cephalometric norm, such as angular measurements, does not contribute significantly to the precise estimation of the MCL. The low coefficient of determination (R² = 0.109) indicates that it explains only 10.9% of the mandibular variability, a performance consistent with previously described for norms derived from Caucasian populations [8,29]. In contrast, the linear variables, ACBL, PCBL and RH, exhibited significant associations and greater predictive capacities (R² = 0.646; 0.533; 0.576, respectively). Our findings align with reports that describe a moderate-strong positive correlation between ACBL and MCL, especially in Class I and Class III malocclusions [10,30] and the observation that a shorter ACBL is usually accompanied by shortened mandibular bodies in Class II [11].

The integration of these three variables in a multivariate model increased the explanatory capacity to R² = 0.869. This increase of ~22% compared with the best individual model confirms that the combination of linear measurements improves diagnostic precision, a finding that is consistent with multivariate and machine learning approaches that have shown greater accuracy and efficiency [2,20]. This cumulative effect can also be interpreted considering the concurrent influence of basal flexion: morphometric studies show that a more closed SA increases the effective length of the body and the ramus, which improves the total explanation of the variance when considered together with linear lengths. This model presented more uniform residuals and a median close to zero, elements that favor its clinical application by minimizing under- or overestimation biases.

Importantly, the angular measurements (saddle, articular, and gonial) did not show significant associations in isolation (Table 2). Only the gonial angle maintained significance in the multivariate model, although with a negative coefficient, a pattern that was previously described as indicative of an inverse interaction between mandibular inclination and body length [13]. This finding reinforces previous observations that individual cranial angles, unlike global basal flexion, present high intra- and inter-observer variability and little stability during growth, thus reducing their prognostic value [12,19].

Regarding the sociodemographic variables, in the full multivariate model, neither age nor sex was significant for MCL in young adults, which is consistent with investigations showing that mandibular morphology remains stable after skeletal maturity [29]. However, significantly longer body and ramus lengths have been described in males in more heterogeneous samples, especially in Class III patterns [10,11], suggesting that the sex effect emerged with larger sample sizes or in differentiated populations. Therefore, we suggest that cranial-base features naturally adjust for gender and age-related differences in the multivariate model.

The graphical evaluation of the residuals revealed that the multivariate model exhibits the lowest dispersion and the fewest outliers. While the cephalometric norm demonstrated low dispersion, the correspondingly low R² value further highlighted its inadequacy as a predictive tool; similar findings were described for “static” versus “floating” norms on the basis of adaptive statistics [6]. This graphical stability supports the daily use of the multivariate model as a screening and planning tool, as has been proposed in floating norm algorithms and in deep learning models aimed at predicting mandibular growth trajectories [14,20].

Limitations and strengths

This study has several limitations that should be considered when interpreting the findings. First, due to its cross-sectional design, this study cannot establish causal or dynamic relationships of mandibular growth over time. Since the measurements were taken at a single moment, it is not possible to assess how the length of the mandibular body varies with development or how the relationships between craniofacial structures might change at different stages of life. Longitudinal studies have shown that the correlation between basal flexion and mandibular growth is accentuated at puberty, so future studies should use time series to capture this phenomenon [14].

Second, the sample used was drawn from a specific population: young adults who attended the orthodontic clinic of a private university in San Luis Potosí, Mexico. Although strict inclusion criteria were used to ensure the homogeneity and quality of the measurements, this selection may limit the generalizability of the findings. The results may not be completely extrapolated to other populations with different characteristics, such as growing adolescents, older adults or individuals with a history of orthodontic treatment, developmental disorders or different ethnic and morphological components. Prior studies have shown that cranial base length and flexion vary across ethnic groups, influencing mandibular morphology [12].

Another relevant limitation, sometimes cited as a limitation by other authors, is related to the use of artificial intelligence for automated cephalometric tracing through the WebCeph® software [23,31-33]. Although this technology offers advantages in terms of efficiency and standardization, the automatic identification of anatomical landmarks can be affected by the quality of the image, the overlapping of structures, the presence of artifacts or individual anatomical variations [22,31,32]. Although the images were carefully selected and obtained with standardized protocols, the possibility of errors in the automatic positioning of reference landmarks cannot be entirely excluded; however, we performed an intra-instrument analysis and ensured high measurement quality, which minimized any potential impact on precision [19]. Additionally, we regard this technology as a methodological strength: its algorithmic workflow greatly increases efficiency, eliminates most operator-dependent subjectivity, and enhances both reproducibility and objectivity in the measurements.

Furthermore, although the statistical model used was robust and validated via a stepwise approach to reduce overfitting, no external validation analyses were performed. That is, the results obtained were not contrasted with a second independent sample or through cross-validation methods, this could limit the generalizability to other clinical settings other clinical contexts. Recent meta-analyses recommend cross-validation for any cephalometric predictive algorithm [20].

The multivariate model developed prioritized the predictive capacity of the selected variables, omitting possible exploratory analyses of complex interactions between variables or moderating effects. Although the main objective of this study was to identify efficient predictors of the length of the mandibular body, future research could delve into more complex models, such as hierarchical regressions or machine learning techniques, that integrate a greater number of clinical, morphometric and contextual variables [22,23].

Even with the benefits of multivariate MCL estimation, situations may arise where the full model is impractical - such as in clinics with limited resources or without access to regression software - ACBL may still serve as a pragmatic first-line proxy. Its utility stems by leveraging its classic one-to-one relationship with mandibular corpus length; nonetheless, it should be combined with additional linear measurements to reduce diagnostic error; in this way, traditional cephalometric norms and the Björk-Jarabak proportion serve as a fundamental instrument, with ABCL providing an easily applicable guide for estimating mandibular length.

Future research

We believe that cephalometric norms will remain a key clinical benchmark for diagnosis; however, as this study demonstrates, assessing cephalometric measurements alongside other craniofacial or dental structures yields a more comprehensive understanding of morphology. For instance, if an individual’s cranial base length exceeds the norm, it is reasonable to expect other measurements to be proportionally higher, and the reverse if they are lower. In this way, cephalometric can be approached from a standpoint of proportional adequacy rather than strict adherence to fixed norms. Moreover, the use of multivariate techniques and the emerging capabilities of artificial intelligence will pave the way for more sophisticated, automated models that enable truly personalized cephalometric analysis.

## Conclusions

Our data strongly suggests that conventional cephalometric norms should no longer be used as a stand-alone diagnostic yardstick tool. Their limited explanatory power and lack of patient-specific tailoring, already highlighted in previous research, make them unreliable for predicting MCL. In contrast, a multivariate equation that combines ACBL, PCBL, RH and Gon angles offers a significantly more accurate and clinically useful estimation. These findings reinforce growing evidence that both the length and flexure of the cranial base exert decisive control over mandibular dimensions.

In clinics with limited resources or no access to regression software, the full multivariate model might not be possible. In such cases, the ACBL can be a useful first step. It has a simple one-to-one relationship with mandibular corpus length. To reduce errors, it should be used with other linear measurements. This way, traditional cephalometric norms and the Björk-Jarabak proportion can still help estimate mandibular length.
